# Mitigating Ecotoxicity Risks of Pesticides on Ornamental Plants Based on Life Cycle Assessment

**DOI:** 10.3390/toxics11040360

**Published:** 2023-04-10

**Authors:** Xinhan Yin, Lei Feng, Yi Gong

**Affiliations:** Key Laboratory of the Three Gorges Reservoir Region’s Eco-Environment, Ministry of Education, Chongqing University, Chongqing 400044, China

**Keywords:** life cycle assessment, pesticides, ecotoxicity, ornamental plants, PestLCI, USEtox

## Abstract

Ornamental plants such as floriculture and nurseries, have become increasingly popular, but their growth relies heavily on the use of many different types of pesticides. The widespread and inefficient use of these pesticides causes environmental pollution and damage to non-target organisms. Despite these impacts, there has been little research conducted on potential agrochemical pollution in the ornamental plant industry. To address this gap, a life cycle assessment (LCA) was conducted to evaluate the pesticide-related freshwater ecotoxicity impact of the US ornamental plant industry in comparison to that of major field crops. The study analyzed 195 pesticide active ingredients used in 15 major ornamental plant and four field crops. Results showed that the freshwater ecotoxicity per area (PAF m^3^ d/ha) of ornamental plants was significantly higher than that of field crops due to the high pesticide intensity (kg/ha) and ecotoxicity of insecticides and fungicides used in floriculture and nurseries. To mitigate environmental stress, minimizing the use of highly toxic pesticides is recommended. A ban on low-dose, high-toxicity pesticides could reduce pesticide-driven ecotoxicity by 34% and 49% for floriculture and nursery plants, respectively. This study is among the first to quantify the pesticide-driven ecotoxicity impacts of horticultural ornamental plants and proposes feasible ways to reduce these impacts, thus making the world more sustainable while still preserving its beauty.

## 1. Introduction

Ornamental plants (i.e., floriculture and nurseries) are highly valued for their spiritual symbolism and emotional significance [1,2]. Over the past decades, the demand for these plants has significantly increased as people increasingly used them for beautification and decoration [3]. The global market for ornamental plants is currently valued at USD 52.3 billion and is projected to grow by over 50% in the next five years [4]. Although ornamental plants deliver significant socio-economic benefits, they also pose significant challenges to the environment [5]. Numerous studies have examined the environmental impacts of ornamental plants, such as their greenhouse gas emissions and impacts on land use and biodiversity [6,7,8,9]. However, their ecological impacts associated with pesticides have yet to be fully explored, primarily due to the lack of national-scale pesticide application data and the complexity involved in conducting ecotoxicity assessments for pesticides [10,11].

Pesticides are crucial for safeguarding ornamental plants from pests, diseases, and weeds and maintaining their appearance [5,12]. However, the widespread and inefficient use of pesticides has led to considerable environmental emissions through leaching and runoff [13]. As a result, soil and water are being contaminated, and non-target organisms, such as beneficial insects and fish, are being poisoned [14,15]. For instance, herbicides such as atrazine can negatively affect the sexual development of amphibians, while neonicotinoid insecticides such as clothianidin can damage the immune systems of bees [16,17,18]. Given the growing demand for ornamental plants in the near future [4], we should take urgent action to mitigate the ecotoxicity impacts of pesticides and promote sustainable horticultural practices.

Life cycle assessment (LCA) is widely used to quantify the cradle-to-grave impacts of products and systems [19]. It consists of four stages: the first stage defines the goals and scope of the study; the second stage is the inventory analysis (LCI) stage; the third stage is the impact assessment (LCIA); and the last stage provides a qualitative or quantitative evaluation to identify potential “hotspots” in the system [20]. The reliability of LCA results mainly depends on the quality of LCI and LCIA models. LCA has been widely applied in the evaluation of ornamental plants. However, current research has focused chiefly on the overall assessment of individual plants or the carbon footprint accounting of flower and nursery plant groups [12,21]. There is a lack of LCA studies that examine the fate and environmental impacts of pesticides in ornamental plants on a larger scale.

One of the major challenges in conducting an LCA of ornamental plant systems is accurately simulating the emissions resulting from pesticide use and coupling them with environmental impact characterization models [22]. In the LCI stage, current studies typically assume that 100% of pesticides enter agricultural soil [23]. However, this simple allocation method ignores important factors such as application methods, crop types, and natural environmental variations that can affect the distribution of pesticide emissions. For example, high temperatures can accelerate the degradation and volatilization of pesticides [24]. In the LCIA stage, characterizing the toxicity of different pesticides is exceptionally complex due to diverse pesticide active ingredients and their heterogeneous ways affecting non-targeted organisms in the environment. Additionally, pesticide ecotoxicity is not solely dominated by toxicity parameters, but also depends on other factors such as application rates, mobility and persistence in the environment, exposure pathways, and biological utilization.

To assess the environmental risks associated with pesticide use in ornamental plants while minimizing impacts on crop production, this study utilized advanced models in the LCA pesticide assessment field—the PestLCI and USEtox models from the OLCA-Pest project [25]. The study collected application data on 106 pesticide active ingredients (AIs) commonly used for 15 ornamental plants in the US, which is the largest consumer of ornamental plants and has readily available pesticide use data [26]. In addition, we collected application data on 119 AIs commonly used for four field crops and compared them with those used in ornamental plants to better illustrate the intensity and risk of pesticide use in ornamental plants. Then, the fate and distribution of different pesticides in each environmental compartment were simulated, and the freshwater ecotoxicity impact of ornamental plants was quantified relative to field crops. Finally, pesticide hotspots in various ornamental plants were identified, and feasible solutions were proposed to reduce the ecotoxicity impacts associated with pesticide use.

## 2. Materials and Methods

### 2.1. Pesticide Usage Data Collection

The study utilized pesticide usage data from the National Agricultural Statistics Service (NASS) Pesticide Use Program. This program aims to monitor and assess the use of pesticides, fertilizers, and other chemicals in crop production by American farmers [27]. To collect this data, NASS employs various methods, including distributing survey questionnaires to farmers and crop growers, working with pesticide manufacturers and sellers to collect sales and usage data, and verifying and supplementing data by monitoring and statistically analyzing pesticide residues in water, soil, and air. In addition, NASS investigates reports from agricultural advisers, state governments, and federal agencies to obtain more comprehensive data. The program surveys pesticide usage data for 2 to 6 crops annually and covers at least 80% of the national planting area for each target crop. Through the Pesticide Use Program, NASS is able to understand pesticide usage in American agricultural production, including information on the types, quantities, frequencies, and methods of pesticide usage. Based on this program, we compiled 24,719 raw data entries and collected application data for 19 crops and 195 AIs, including 15 major ornamental plants and four field crops, and is exhaustively explicated in Table 1.

### 2.2. Ecotoxicity Assessment of Pesticide Based on LCA

This study utilized the pesticide assessment method recommended by the OLCA-Pest project, which aimed to provide a pesticide emission and environmental impact assessment method under the LCA framework to support agricultural life cycle assessment. The project provides data and models covering different pesticides, which can be used to evaluate the impact of pesticide use on ecosystems. The LCI phase employed the network-based PestLCI Consensus model to simulate the emission fractions of different pesticides in each compartment after entering the environment. The LCIA phase utilized the science-based consensus model USEtox, recognized by the United Nations Environment Programme’s Life Cycle Initiative, to characterize the ecotoxicological impacts of pesticides.

To evaluate the ecotoxicity impact of pesticide AIs according to the OLCA-Pest project’s definition, the ecotoxicity impact score method is utilized, which involves the following three steps [25]:

1. Conducting an LCI analysis on the pesticide active ingredients by taking into account the application method, timing, quality, and crop growth stage of the pesticide to determine the proportion of pesticide active ingredient emissions in the field and off the field.
2. Assessing the ecotoxicity characterization of the pesticide active ingredient in the LCIA stage.
3. Establishing a correlation between the environmental compartment emission distribution in the LCI and LCIA stages and the toxicity characterization of the pesticide in different environmental compartments.

The freshwater ecotoxicity impact score (IS) per unit area of crops can be described as follows:

(1) $$\begin{array}{c} IS=\overset{}{\underset{p,\;\;c}{\sum }}\left(m_{emi,p,c}\times CF_{p,c}\right)\\ m_{emi,p,c}=m_{app,p}\times f_{c} \end{array}$$

where *IS (*$PAFm^{3}d$*)* is the impact score of all pesticide environmental toxicities, $m_{emi,p,c}$ (kg) is the total emitted mass of pesticide *p* into environmental compartment *c*, and $m_{app,p}$ (kg) is the total mass of pesticide *p* applied to farmland.

#### 2.2.1. Goals, Functional Unit, and System Boundary

As previously mentioned, the first step in LCA research is defining the goal and scope. The objective of this study is to assess the ecotoxicological impacts of pesticides on ornamental plants in the US. According to ISO (2006b), the determination of a functional unit (FU) is the foundation for conducting an LCA study, and in this study, the FU is defined as ornamental plants grown on one hectare of farmland. In LCA studies, the system boundary illustrates all the operations, resource inputs, and outputs of the system. In this study, the input is the amount of pesticide applied per unit area of farmland, and the output is the emissions of the pesticide to various environmental compartments.

#### 2.2.2. Life Cycle Inventory: Tracking the Environmental Fate of Pesticides

The emission fractions of various pesticides in different compartments upon entering the environment were estimated using the PestLCI Consensus v.1.0 model. The model was developed by Dijkman et al. in 2006 and updated by Fantke et al. in 2017 [28,29]. It is a modular model that calculates the proportion of pesticide emissions to air, soil, surface water, and groundwater based on specific information about pesticide application scenarios, such as application method, crop type, growth stage during application, soil and climate data in the treated area, and physical and chemical properties of pesticide active ingredients.

Compared to other life cycle inventory assessment methods for pesticides, this model better reflects the changes in pesticide emission fractions caused by differences in pesticide characteristics, soil and climate conditions, and pest management strategies adopted by farmers.

The model estimates pesticide emissions based on two distributions. The first considers the initial processes after pesticide application, such as drift loss, leaf litter, and soil deposition. The second integrates processes that occur on crop leaves (such as degradation, volatilization, and plant uptake) and on soil surfaces (such as volatilization, degradation, leaching, and runoff). Based on the results of the second distribution, the proportions of pesticide emissions to soil, air, surface water, and groundwater, as well as plant compartments, are estimated. In national-scale studies, the OLCA-Pest project recommends using an initial emission allocation fraction [25]. Therefore, this study did not consider the second tier of the model’s emission allocation.

The model generated distribution data for four environmental compartments ($f_{c}$): the initial emission fractions to air ($f_{air}$), off-field surfaces ($f_{dep}$), field soil ($f_{field/soil}$) and field crop ($f_{field/crop}$). With the following calculation formula [24]:

(2) $$\begin{array}{c} 1=f_{air}+f_{dep}+f_{field/soil}+f_{field/crop}\\ f_{field}=f_{field/soil}+f_{field/crop} \end{array}$$

where $f_{c}$ is the emission fraction of pesticides into environmental compartment *c*, and $f_{air}$ can be directly determined by the crop and pesticide application method.

(3) $$\begin{array}{c} f_{field/crop}=f_{field}\times f_{intercept,crop}\\ f_{field/soil}=f_{field}\times \left(1-f_{intercept,crop}\right)\; \end{array}$$

where $f_{intercept,crop}$ is the fraction of pesticide intercepted by crop foliage, the value can be obtained from the fraction of field area covered by crop foliage. More detailed calculation procedures can be found in the calculation files of the PestLCI Consensus model [25].

#### 2.2.3. Life Cycle Impact Assessment: Assessing the Ecotoxicity of Pesticides

The ecotoxicological effects of pesticides were evaluated using the USEtox v 2.12 model (https://usetox.org, accessed on 26 February 2023), which is scientifically endorsed and part of the United Nations Environment Programme’s Life Cycle Initiative [30]. This model quantifies the entire process of pesticide AI, from entering the environment to producing aquatic ecotoxicity, by linking the three processes of environmental fate, exposure, and toxicity effects. For ecotoxicity assessment, USEtox calculates the proportion of species affected by AIs in aquatic ecosystems based on concentration-response curves obtained from pathological studies, quantifying the toxic impact. The USEtox model can be adjusted and modified for different application scenarios, including consideration of different exposure pathways, population groups, and ecological environments. Its application can provide policymakers and decision-makers with a scientific basis for chemical management. The model calculates the characterization factor (*CF*) for the ecotoxicity of active ingredients of pesticides that enter the environment, which is used to quantify the toxic impact of chemicals on human health and ecosystems. The CF for ecotoxicity can be calculated using the following formula:

(4) $$CF_{p,c}=FF_{p,c}\times XF_{p,c}\times EF_{p}\;$$

where $CF_{p,c}$ (kg) is the environmental toxicity of pesticide *p* into environmental compartment c, $FF_{p,c}$ is fate factors, $XF_{p,c}$ is exposure factors, $EF_{p}$ is effect factors. The specific parameters and solving process for the three factors can be obtained in the calculation files of the USEtox model.

The environmental fate factor (*FF*) represents the length of time that AI remains in the environment, while the exposure factor (*XF*) represents the amount of bioavailable AI. The effect factor (*EF*) quantifies the toxicity of AI to aquatic organisms by measuring the proportion of species affected by changes in AI unit concentration. A higher value of the effect factor *EF* indicates a greater impact of AI on organisms. The environmental fate and exposure of different chemicals are determined by their physicochemical properties and environmental factors. Finally, the USEtox model calculates the toxic impact of AI released into the air, water, and soil on aquatic ecosystems by taking into account these factors.

The recommended method from the OLCA-Pest project was used to link the output of the PestLCI Consensus model in the LCI phase with the USEtox model in the LCIA phase. This approach enables a comprehensive evaluation of the potential freshwater toxicity impacts of pesticides by avoiding redundant calculations in quantifying emission proportions and impact assessment fate modeling.

The study focuses on two scales following pesticide application: spatial and temporal. At the spatial scale, the field and its surroundings were concentrated in the LCI phase. In the LCIA phase, the analysis was expanded to the national land scale to evaluate the fate distribution process of pesticides in the environment. At the temporal scale, the distribution of pesticides within minutes to days after the application was examined in the LCI phase, aiming to understand their short-term effects on the local ecosystem. In the LCIA phase, the focus was on the long-term impacts of pesticide use on the environment, accurately assessing their bioaccumulation potential and ecotoxicity.

The amount of pesticide application was allocated into four emission scores, including air, crop surfaces, field soil, and off-site surfaces, using the PestLCI Consensus model. The off-site surfaces were further divided into agricultural soil, natural soil (including urban areas), and freshwater environmental parts based on the share of each land use type and water surface in a given area. The land type data of the US in the FAO database were used to assign 16%, 77%, and 7% of the area to agricultural soil, natural soil (including urban areas), and freshwater surface, respectively. These data were then linked to the USEtox model [31].

**Figure 1:**
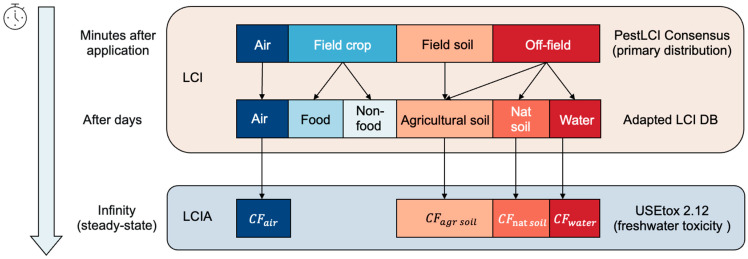
The point of connection between LCI and LCIA in the context of pesticide application for crop production.

The USEtox model generates four freshwater ecotoxicity characterization factors for four environmental compartments: rural air, field soil, natural soil, and freshwater. The relationship between the distribution of the PestLCI Consensus model environmental compartments and the ecotoxicity characterization in the LCIA phase is depicted in the Figure 1.

The air compartment in PestLCI Consensus is mapped to the ecotoxicity characterization of rural air in the USEtox model; the field soil compartment and the compartment that drifts to off-site field soil in PestLCI Consensus are mapped to the ecotoxicity characterization of agricultural soil in the USEtox model; the compartment that drifts to off-site natural soil in PestLCI Consensus is mapped to the ecotoxicity characterization of natural soil in the USEtox model; and the compartment that drifts to off-site freshwater in PestLCI Consensus is mapped to the ecotoxicity characterization of water bodies in the USEtox model.

#### 2.2.4. Hotspot Pesticide Identification

Data on pesticides that are banned in Europe and the US were examined during the investigation, revealing 367 pesticides that are prohibited in Europe but still permitted for use in the US. Through a comparative analysis of these data in combination with information regarding horticultural pesticides, 35 pesticides were identified as “hotspots.” To mitigate any negative effects on crop quality and yield, six specific hotspot pesticides that are commonly used in flower and nursery crops, have high levels of toxicity and are applied at low rates were homed in on. In order to explore the potential for reducing toxicity without adversely affecting crop yield, a series of simulation optimization experiments were conducted, setting the application rates of these six pesticides to zero.

## 3. Result

### 3.1. Pesticide Usage and Ecotoxicity

**Figure 2:**
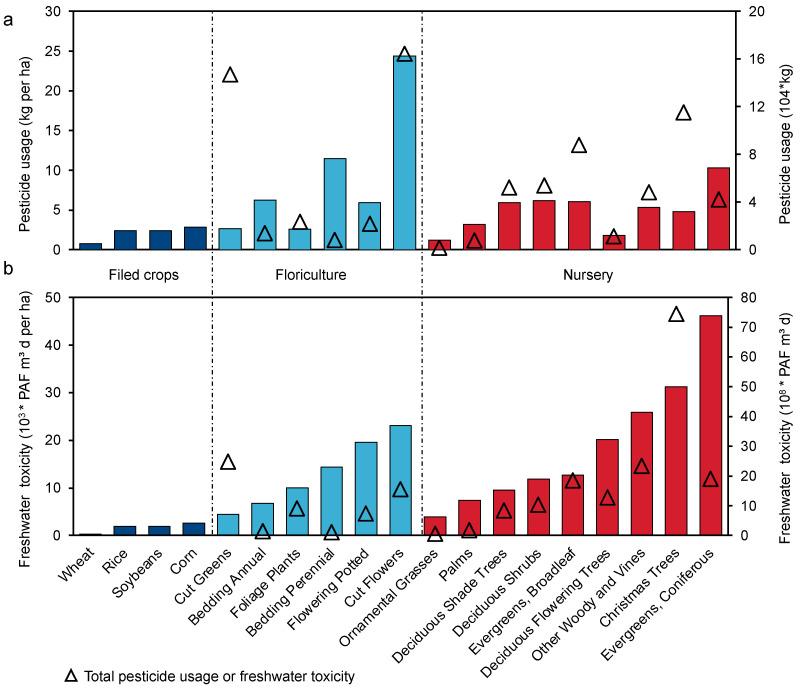
Pesticides usage (**a**) and ecotoxicity (**b**) across floriculture, nursery, and field crops. The bars represent the pesticide usage or ecotoxicity per ha of cropland, while the triangles represent the total pesticide usage or ecotoxicity of crops.

When it comes to pesticide use, there are significant differences between floriculture, nursery, and field crops, as shown in Figure 2a. The ornamental sector, which includes cut flowers, cut greens, and Christmas trees, has a pesticide application rate of over 10 tons due to high market demand. Among these crops, cut flowers have the highest pesticide use per unit area, reaching 24.4 kg/ha, which is twice that of bedding perennials, nine times higher than corn, and 33 times higher than wheat. Generally, except for some individual crops, ornamental horticultural crops have higher pesticide use per unit area than field crops. There is little difference in pesticide use per unit area between flowers and nursery crops, except for cut flowers.

Significant disparities in pesticide use and environmental toxicity between horticultural and field crops were observed, as depicted in Figure 2b. Christmas trees, cut greens, and cut flowers have higher freshwater toxicity due to their usage levels. However, evergreen conifers have the highest freshwater toxicity per unit area, with a value of 46,200 PAF m^3^ d/ha, which is nine times that of corn and 80 times that of wheat. The environmental toxicity of cut flowers and Christmas trees also exceeds 20,000 PAF m^3^ d/ha. Generally, nursery plants have higher pesticide freshwater toxicity than flowering plants, and both show significantly higher levels than field crops. This indicates that the environmental impact of flowers and nursery crops is more toxic compared to that of field crops.

### 3.2. Variation of Pesticide Usage and Ecotoxicity across Crop Types

**Figure 3:**
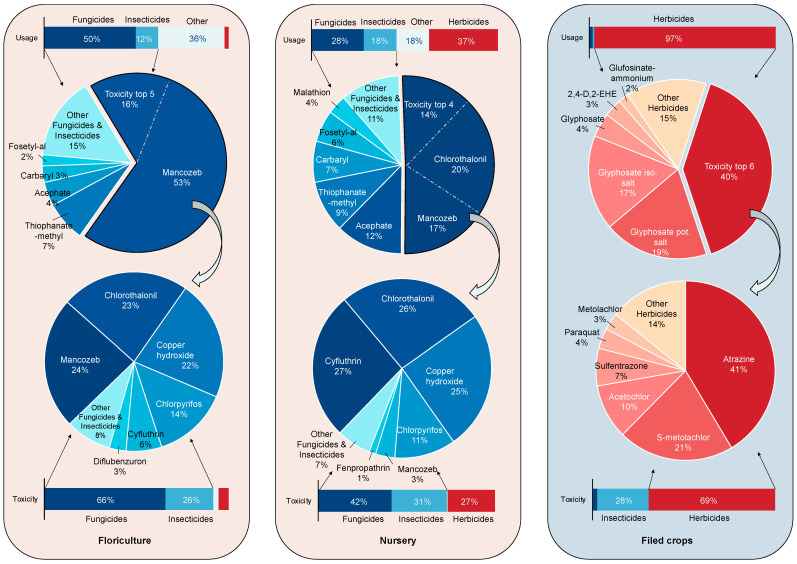
Pesticides usage and ecotoxicity variation analysis.

The research findings suggest that floriculture and nursery crops have a higher demand for insecticides and disinfectants compared to field crops, as shown in Figure 3. In terms of pesticide usage, insecticides, and fungicides makeup 66% of pesticides used in floriculture crops and 46% of pesticides used in nursery crops. In contrast, herbicides account for 97% of pesticides used in field crops, while insecticides and fungicides are less than 3%. The higher proportion of insecticides and fungicides used in ornamental and nursery crops corresponds to a relatively higher proportion of toxicity. From an environmental toxicity perspective, insecticides and fungicides in ornamental plants account for 92% of pesticide toxicity, while the proportion for nursery crops is 64%. In contrast, herbicides contribute to 69% of the total pesticide toxicity in field crops. This is primarily due to the need for a more stringent growing environment in floriculture and nursery crops, which requires greater use of insecticides and fungicides to ensure plant growth and appearance quality. Additionally, these crops typically have a higher sales value and shorter growth cycle, so producers take more preventative measures to ensure plant health and appearance quality.

### 3.3. Hotspot Identification

In order to better develop pesticide use strategies and protect the ecological environment for farmers, pesticide manufacturers, and agricultural researchers, it is important to identify pesticide hotspots. Pesticide hotspots refer to situations where there is a high ecotoxicity risk after the use of pesticides in a certain crop or region. Compared to field crops, ornamental plants demand a higher standard for product appearance and rely more heavily on pesticides to avoid damage to their leaves or flowers, resulting in higher pesticide intensity and risk for ornamental plants. Therefore, identifying hotspots for low-dose, high-toxicity pesticides is crucial in ornamental plant cultivation. By recognizing these hotspots, farmers and pesticide manufacturers can determine which pesticides to avoid, thereby minimizing ecological risks while ensuring maximum yield and quality of ornamental plants.

**Figure 4:**
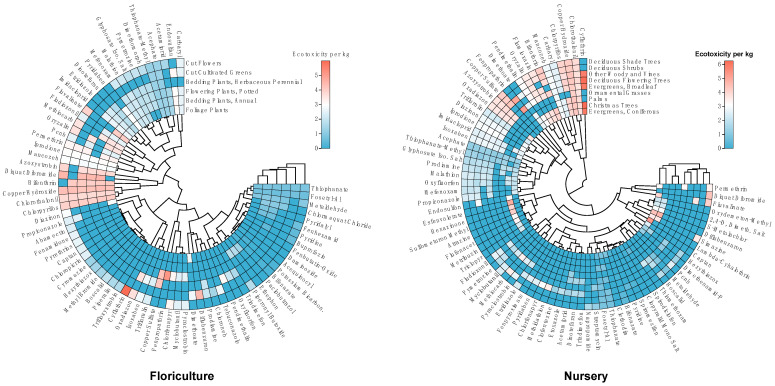
Identification of crop–pesticide hotspots.

Figure 4 illustrates the ecotoxicity of different pesticides in terms of unit dose across various crops. Specifically, cut flowers using cyfluthrin and diquat dibromide, as well as potted flowers using fenpropathrin, exhibit relatively high ecotoxicity per unit dose in the case of flower crops. Similarly, nursery crops using cyfluthrin and fenpropathrin also exhibit high ecotoxicity per unit dose. Thus, it is essential to exercise caution when using these pesticides to avoid adverse effects on the ecological environment. In summary, by understanding the information on crop–pesticide hotspots, farmers, pesticide manufacturers, and agricultural researchers can more effectively protect the health of crops and the environment.

### 3.4. Mitigation of Pesticide Ecotoxicity Impact

**Figure 5:**
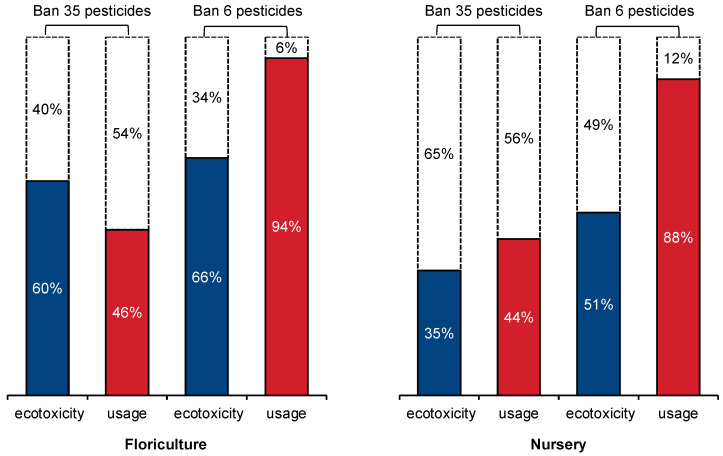
The potency of mitigation of pesticide ecotoxicity. The dashed boxes represent the reduction in ecotoxicity or dosage through banning hotspot pesticides, and the solid boxes represent the usage or ecotoxicity of remaining pesticides.

Europe is clearly ahead of the US in terms of banning harmful pesticides. The US ornamental plant pesticide application database shows that 35 pesticides banned in Europe are still being used in the US ornamental plant industry. Prohibiting these 35 pesticides could reduce the ecological toxicity of flowers by 40% and nurseries by 65%, as depicted in Figure 5. However, banning these 35 pesticides altogether could significantly impact the quality and yield of horticultural plants due to the reduction in pesticide doses. To address this, hotspot pesticide identification was combined with the selection of six pesticides with low doses but high toxicity for simulation optimization in floriculture and nursery plants. These six pesticides for floriculture plants are chlorothalonil, cyfluthrin, diquat dibromide, bifenthrin, fenpropathrin, and oxadiazon, accounting for only 6% of total pesticide use but contributing to 34% of the total toxicity. The six pesticides chosen for nursery plants include cyfluthrin, chlorothalonil, hexazinone, fenpropathrin, bifenthrin, and diquat dibromide, accounting for only 12% of total pesticide use but contributing to 49% of the total toxicity. A transition period is necessary for comprehensively banning high-risk pesticides to ensure farmers can adapt to new agricultural production methods without significant impacts on their yield and quality.

## 4. Discussion

According to the study, the ecotoxicity impact of pesticides on ornamental plants is greater than that of field crops. This is because ornamental plants have a higher economic value and stricter appearance requirements, which results in the application of high-toxicity insecticides and fungicides to reduce pests and diseases. Although these pesticides have ensured the high quality and profitability of ornamental plants, they have also led to a significant increase in environmental pressures.

There are diverse ways to reduce pesticide-induced environmental impact, a potentially feasible approach being to reduce high-toxicity low-dose pesticides, as we evaluated. While this approach has considerable potential to mitigate ecotoxicity from hotspot pesticides, the remaining pesticides can significantly damage the environment. Further damage reduction needs more effort. Firstly, a more scientific pesticide evaluation system is needed [22]. Researchers need to further improve the life cycle assessment system for pesticides, including pesticide emission and ecotoxicity characterization, which would help growers choose relatively efficient and safe pesticides [32]. Secondly, pesticide utilization needs to be improved, and the spread of pesticides into the environment needs to be suppressed. With the view on usage, governments should strengthen public education on methods of pesticide application (e.g., following the instructions on pesticide labels, choosing suitable application methods, and reducing pesticide drift) [33,34]. From a technical view, nano-pesticides need to be developed [35,36]. Compared to traditional pesticides, nano-pesticides can package and deliver AIs in different response ways (such as controlled, targeted, and synchronous), which may improve the effectiveness and efficiency of pesticides [37]. Finally, non-pesticide technologies need to be developed, such as resorting to natural predators to control pests or planting disease-resistant crops with modified gene [38,39,40,41].

LCA is widely used to assess the environmental impacts of agricultural practices. In this study, the ecotoxicity risk of pesticides for ornamental plants in the USA was assessed on a national scale using the LCA approach. The advantage of using LCA is that it is holistic and standardized. By analyzing the fate processes of different pesticides in the environment and their toxicity characterization, LCA allows for the identification of hotspot pesticides, which enables stakeholders to develop targeted environmental improvement measures that ultimately reduce pesticide risks in agricultural practices.

However, there are some limitations to the use of LCA in the agricultural sector. One limitation is the availability of data. LCA requires large amounts of data that may not always be readily available for agricultural practices, especially in developing countries where data collection may be limited. Our study collected pesticide usage data for ornamental plants in the US. However, the NASS database may contain hidden values, resulting in possible underestimations of the actual pesticide dosage and environmental toxicity. Nonetheless, these possible underestimations only reinforce the significant adverse environmental impacts resulting from pesticide use and the need to address and mitigate these impacts. While our study focused on the US situation, developing countries rely more heavily on pesticide use for growing ornamental plants due to higher production and lower efficiency [12,33,42,43]. Unfortunately, these developing countries have limited pesticide use data, which makes it challenging to comprehensively evaluate the magnitude of national pesticide applications and damages. Although some studies attempted to simulate pesticide use at the national or subnational scale, these simulations are not perfect and may introduce biases to future research on pesticide-related risks. Therefore, cooperation between governments, industry departments, and scientific research institutions is crucial for pesticide use data collection and monitoring beyond current levels.

Secondly, the scope of this study is limited to the environmental impacts of pesticides and does not consider the damage to human health through various pesticide exposures. Pesticides can be spread into the air after application, negatively affecting the respiratory systems of farmers and nearby residents [44,45,46]. Additionally, pesticide residues on crops can end up in humans through inhaling or directly consuming the edible parts of flowers. Thus, it is necessary to further expand the scope of the to involve the human toxicity of pesticides to make the assessment system more comprehensive.

In conclusion, while LCA is a valuable tool for assessing the environmental impact of agricultural practices, it is important to consider its limitations and use the results in conjunction with other information to make informed decisions. By doing so, stakeholders can develop more sustainable agricultural practices that balance environmental, social, and economic factors.

## 5. Conclusions

The ecotoxicity risks of pesticide use on ornamental horticultural crops were quantified in the study from a macro perspective, and a solution was proposed based on the agricultural life cycle assessment. A pesticide application database was established for 15 major ornamental plants, and a comprehensive assessment framework for the environmental impact of pesticides across multiple crops and ingredients over their entire life cycles was constructed by integrating pesticide environmental emission models and toxicity characterization models. The results indicated that the pesticide application and freshwater ecotoxicity for producing per unit of ornamental plants were significantly higher compared to field crops. To reduce the ecotoxicity of pesticides, a priority pollutant screening and ranking method was used, combined with a pesticide prohibition list, to identify pesticide hotspots that have low dose but high toxicity. Banning the identified hotspots while minimizing the impact on crop production can reduce the ecotoxicity of pesticides on floriculture by 34% and nurseries by 49%. The study provides a new quantitative analysis framework for assessing strategies to reduce pesticide-driven ecotoxicity while minimizing influences on crop production.

## Figures and Tables

**Table 1:** Essential details from the AIs database for plants or crops.

Category	Plants or Crops	Number of AIs
Floriculture	Cut Greens	20
Floriculture	Bedding Annual	39
Floriculture	Foliage Plants	32
Floriculture	Bedding Perennial	34
Floriculture	Flowering Potted	40
Floriculture	Cut Flowers	52
Nursery	Ornamental Grasses	8
Nursery	Palms	12
Nursery	Deciduous Shade Trees	36
Nursery	Deciduous Shrubs	38
Nursery	Evergreens, Broadleaf	45
Nursery	Deciduous Flowering Trees	26
Nursery	Other Woody and Vines	46
Nursery	Christmas Trees	32
Nursery	Evergreens, Coniferous	41
Field crops	Wheat	53
Field crops	Rice	33
Field crops	Soybeans	69
Field crops	Corn	56

## Data Availability

The data presented in this study are available on request from the corresponding author.
